# The integrative role of parenting styles and parental involvement in young children’s science problem-solving skills

**DOI:** 10.3389/fpsyg.2023.1096846

**Published:** 2023-06-12

**Authors:** Xunyi Lin, Weipeng Yang, Wanlin Xie, Hui Li

**Affiliations:** ^1^College of Education, Fujian Normal University, Fuzhou, China; ^2^Department of Early Childhood Education, Hong Kong Institute of Education, Tai Po, Hong Kong SAR, China; ^3^Shanghai Institute of Early Childhood Education, Shanghai Normal University, Shanghai, China

**Keywords:** authoritative parenting style, authoritarian parenting style, parental involvement, science problem-solving skills, bidirectional analysis

## Abstract

**Introduction:**

How parents encourage and engage young children to learn science and solve scientific problems remains an understudied issue. Parenting styles have been widely studied and found to be associated with children’s various developmental outcomes. However, there is a dearth of research linking parenting styles to early science skills which build from both cognitive and social abilities. This cross-sectional study intended to pilot test a mediation model of parental involvement in the relationship between parenting styles and children’s science problem-solving skills.

**Methods:**

A total of 226 children (*M* = 62.10 months, SD = 4.14, 108 girls) and their parents was recruited from five kindergartens in Fuzhou in China by adopting stratified random sampling. All parents completed the Demographics Questionnaire, the Parenting Style and Dimension Questionnaire, and the Chinese Early Parental Involvement Scale. Each child was tested with the Picture Problem Solving Task. Pearson’s correlation analysis and intermediary effect analysis were conducted using IBM SPSS 25 in data analysis.

**Results and discussion:**

Parental involvement had a significant mediating effect in the bidirectional associations between parenting styles and children’s science problem-solving skills. The findings suggested that children with higher science problem-solving skills were likely to be raised by parents who were employing a flexible (i.e., authoritative) parenting style and had more involvement in children’s formal and informal learning environments, while children’s higher levels of science problem-solving skills predicted a higher level of parental involvement and a more flexible parenting style.

## 1. Introduction

Young children use science skills such as seeking information and solving problems to explore and interact with the physical world around them (Fusaro and Smith, 2018). Although children are naturally ‘little scientists,’ adult educators such as parents play a critical role in providing opportunities for them to wonder and explore the surrounding world (Kewalramani et al., 2022). Parenting styles have been widely studied by linking different styles to children’s social skills (Rinaldi and Howe, 2012), behavioral problems (Braza et al., 2015), cognitive skills (Rudasill et al., 2013), and early academic skills (Roopnarine et al., 2006). However, there is a dearth of research on the relationships between parenting styles and children’s early science skills such as science-relevant problem solving. Children’s skills of solving science problems build from both cognitive and social abilities, and provide the required foundation for formal schooling and general problem solving (Fusaro and Smith, 2018). Given that an increasing emphasis has been placed on early science as the core of STEM (Science, Technology, Engineering, and Mathematics) learning experiences for young children (McClure et al., 2017), examining the relationship between parents’ role and children’s science skills is a significant knowledge gap to be filled. Moreover, it is crucial to examine the mechanism underlying the possible links from parenting styles to children’s science problem-solving skills, which could shed light on ways to enhance parent practices that support children’s science skills.

### 1.1. Science problem-solving skills in the early years

Science skills are defined as the use of science knowledge and understanding within science-relevant problem-solving situations (Fusaro and Smith, 2018); therefore, they are also named science problem-solving skills or scientific inquiry skills (Lin et al., 2020a). As emergent STEM skills, young children’s science skills are closely related to reasoning and problem-solving abilities, which are not less important than academic skills such as numeracy and literacy. In those circumstances of effectively solving science problems, children need to be able to integrate and organize both visual-motor and symbolic reasoning skills with flexibility and creativity (Karpov, 2003). Tolmie et al. (2016) further conceptualize early science skills to include observation skills, descriptive and explanatory skills, and reasoning and hypothesis-testing skills.

Concerning the relations between parenting and early science skills, to the best of our knowledge, there is only one study revealing that parents’ home-based involvement in science education could predict their children’s general science knowledge at kindergarten entry (Stylianides and Stylianides, 2011). However, less valid measurements with secondary data were used in Stylianides and Stylianides’s (2011) study to examine the complex construct of parental involvement and children’s science learning outcomes, thus leaving the nuanced role of parenting in children’s science skills obscured. Therefore, we extended the scope of literature review to cover research on generic child developmental outcomes and their relation to parenting practices.

### 1.2. Parenting styles and child development

Parenting context plays a vital role in shaping children’s cognitive, behavioral and academic outcomes (Darling and Steinberg, 1993; Pinquart, 2016; Augustine and Stifter, 2019). An extensive body of studies have examined the influence of parenting styles and parental involvement on children’s educational and developmental outcomes, especially those school-aged students (see reviews by Masud et al., 2015; Pinquart, 2016; Sangawi et al., 2018).

Parenting styles, or child-rearing styles, refer to the typologies of emotional climate as a context in which parents raise their children (Darling and Steinberg, 1993), which have been characterized by parents’ commitment and balance in terms of responsiveness and demandingness (Baumrind, 1991). As categorized by Baumrind (1991), authoritative parents are both responsive and demanding, while unengaged parents are low in both responsiveness and demandingness. Authoritarian parents, instead, are unbalanced in the two dimensions, with much more demandingness than responsiveness, leading to tight control and little freedom for the children. Specifically, Robinson et al. (1995) described authoritative parenting with characteristics such as high warmth and joy, clear communication of expectations, and democratic participation, while authoritarian parenting might be featured with high control, verbal hostility, restrictiveness and even punitive discipline.

Parenting styles influence child development by providing an environment (both physical and mental) for children to learn and socialize. In general, authoritative parenting is associated with positive developmental outcomes in childhood and adolescence, while authoritarian parenting predicts negative consequences (e.g., problem behaviors). For instance, Hosokawa and Katsura (2019) revealed the negative relationship between the authoritative parenting style and children’s behavior problems. Rinaldi and Howe (2012) also found the authoritative parenting style predicted more adaptive child behaviors, while the authoritarian parenting style was associated with both externalizing and internalizing child behaviors. Based on a longitudinal research project, Baumrind et al. (2010) revealed that adolescents whose parents were more authoritative at their preschool ages tended to be more competent and better adjusted as compared to their counterparts whose parents were classified as authoritarian.

### 1.3. Parental involvement and child development

Parental involvement is a multifaceted construct that subsumes a wide range of parenting practices and parents’ involvement behaviors (Fan and Chen, 2001), including parents’ school-based involvement such as volunteering (e.g., Jeon et al., 2020), parents’ communication and collaboration with teachers (e.g., Murray et al., 2015), and parents’ home-based involvement entailing interactions between parents and their child (e.g., Giallo et al., 2013). Parental involvement is a robust predictor of preschoolers’ school readiness (Lau et al., 2011; Barnett et al., 2020; Jeon et al., 2020) and school-aged students’ academic achievement (Castro et al., 2015; Ma et al., 2016; Boonk et al., 2018; Tan et al., 2020), which has been explained to function through mechanisms such as modeling, reinforcement, and instruction (Hoover-Dempsey and Sandler, 1995). Yet, there is a lack of studies that have included science problem-solving skills or achievement as part of the consequences associated with parental involvement in early childhood. Relations between parental involvement and students’ science achievement were only explored in formal schooling contexts instead of early childhood education (e.g., Liou et al., 2019). In today’s digital era, whether parental involvement can predict young children’s science skills as the core of STEM educational outcomes remains understudied.

### 1.4. The integrative role of parenting styles and parental involvement in child development

Kong and Yasmin (2022) revealed that parents’ characteristics such as their sense of educational efficacy and level of education were positively associated with parental involvement in early childhood education, especially the involvement in home activities with their children. Yet, parenting styles were not included as an influencing factor in their study. In Baumrind et al.’s (2010) longitudinal study, the effect of parenting styles on children’s long-term outcomes was partially attributed to parents’ practices at children’s preschool age, which could be either coercive or confrontational. As a combination of these findings, we assume that parenting styles might affect early childhood development *via* the mediating role of parental involvement. This assumption was partially confirmed in Roopnarine et al.’s (2006) investigation among young children of Caribbean immigrants in the US. As revealed in this study, the authoritarian parenting style was negatively related to children’s academic skills such as receptive skills and vocabulary, while the authoritative parenting style was positively associated with children’s social behaviors (Roopnarine et al., 2006). Meanwhile, parental involvement such as parent-school contacts and parent–child academic interactions was positively related to both academic skills and social behaviors among their children (Roopnarine et al., 2006). However, associations between various parenting styles and parental involvement remained unanswered in this study. It is possible that different parenting styles would lead to varying levels of parental involvement, which together would influence child outcomes. Given the potential interconnections among these parenting factors that could impact child development, the indirect relation from parenting styles to child outcomes (science skills in this study) was of interest in our study.

### 1.5. The reverse effect of children’s learning and developmental outcomes on parenting practices

There are an extensive body of studies on (a) how children’s temperamental characteristics affect parenting practices (e.g., Laukkanen et al., 2014), and (b) evocative effects of child behaviors on parenting (e.g., Shewark et al., 2021). This line of research supports theories that are grounded in bidirectional person (child)-environment (parenting) dynamics such as the transactional model (Sameroff and Mackenzie, 2003). However, there is no research on how children’s science-relevant learning and developmental outcomes may affect parenting practices. Only a few studies have generally examined how child behaviors or well-being may impact on their parenting knowledge and practices. For instance, Shewark et al. (2021) investigated 561 adopted children and their parents and found that child anger would significantly increase parents’ hostility. Dawson-McClure et al. (2015) conducted a 13-week intervention for promoting children’s learning and well-being, which in turn resulted in increased parenting knowledge and parental involvement. In China, Liu et al. (2018) found that primary school students’ early self-regulation negatively predicted later authoritarian parenting through a pathway of academic achievement. However, none of these studies specifically investigate the effect of children’s science-relevant performance on parenting.

Theoretically, child performance may provide the direct feedback on parents’ attempts in educating their children. Children’s optimal development may also enhance parents’ positive attitudes toward their role and reduce parenting stress. Children’s progress in learning would also feed back to their parents’ preferences in determining how to interact with their children in everyday lives, as well as the extent to get involved in children’s learning experiences. These assumptions and ideas about parent–child relations have been supported by overarching conceptual frameworks for understanding dynamics in parenting practices (Kuczynski, 2003). However, there is a lack of evidence testing these theoretical claims, which could significantly increase our understanding of the intersection between parenting and child development.

The present study was conceived using the following strands of theories and evidence. First, an integrative model of parenting style as context (Darling and Steinberg, 1993), suggests that parenting styles would moderate the effects of parenting practices on child development, with evidence revealing that parenting styles encouraging two-way exchanges and child autonomy would predict positive child outcomes (e.g., Baumrind et al., 2010; Rinaldi and Howe, 2012). Moreover, a sociocultural view on child learning and development (Vygotsky, 1962, 1978), suggests that the family context contributes to children’s persistence in asking questions and seeking information. This relates to the extent to which a parent encourages and responds positively to their questioning and exploration (Vandermaas-Peeler et al., 2017, 2018), as well as the meaningfulness of parent–child interaction and communication (Yu et al., 2019). It is worth noting that questioning provides a basis for children’s science-relevant reasoning, problem solving, and cognitive development (Gillies et al., 2012; Fusaro and Smith, 2018).

Despite the well-documented relationships between parenting styles and child outcomes, and between parental involvement and child outcomes, associations between parenting styles, parental involvement, and children’s science-relevant learning outcomes are not directly researched. In order to further understand the mechanism underlying the interaction of parenting styles and the development of child science skills. The present study intends to examine whether the relationships between parenting styles and children’s early science skills are mediated by parental involvement, and whether the mediating effect of parental involvement is significant bidirectionally. Accordingly, specific hypotheses tested are as follows:

*H1*: Parental involvement would mediate the relationship from authoritative (H1a) and authoritarian (H1b) parenting styles to children’s science problem-solving skills.

*H2*: Parental involvement would mediate the relationship from children’s science problem-solving skills to the authoritative (H2a) and authoritarian (H2b) parenting styles.

## 2. Materials and methods

A cross-sectional study design was chosen for this research as it allowed us to collect data from a single point in time, which is useful for investigating relationships between variables that are not expected to change over time (Wang and Cheng, 2020). This design is particularly appropriate for our research questions, which aims to explore the relationship between parental involvement and children’s science skills. By collecting data from a large and diverse sample of participants at a single point in time, we were able to gain a snapshot of the current state of children’s science skills and their parents’ involvement and parenting styles in our population of interest. Additionally, a cross-sectional design allows for efficient data collection and analysis, making it a practical and cost-effective choice for our study.

### 2.1. Participants

Stratified random sampling procedure were adopted to select five kindergartens from different regions (urban/rural) and different levels in line with the local Kindergarten Rating Assessment Program in Fuzhou, a coastal city in south-eastern China. A total of 226 children (*M* = 62.10 months, SD = 4.14) and their parents participated in this cross-sectional study. Family socioeconomic status (SES) varied across the selected kindergartens, as indicated by the differences in parent education, occupation, and household income. Research permission was sought from the principals of the five preschools, after which parents of 5-year-old children were then recruited through a letter explaining the purpose of the study and requesting their consent. The data gathering process was conducted over a period of one month, during which participants were recruited and data was collected through a series of questionnaire surveys and child assessments. Table 1 shows the demographic characteristics of the participating children and parents.

**Table 1:** Demographic data for participants (parents and children).

Demographic characteristics	
Child age in month (*M* ± SD)	62.10 ± 4.14
Child gender	
Female	108 (47.8)
Male	118 (52.2)
Father education	
High school and below	88 (38.9)
Associated degree	51 (22.6)
Bachelor degree	68 (30.1)
Master degree and above	19 (8.4)
Mother education	
High school and below	96 (42.5)
Associated degree	39 (17.2)
Bachelor degree	80 (35.4)
Master degree and above	11 (4.9)
Father vocation	
Semi-technical and technical worker	38 (16.8)
Semi-professional and public servant	97 (42.9)
Professional and officer	81 (35.8)
High-level professional and administrator	10 (4.4)
Mother vocation	
Semi-technical and technical worker	77 (34.1)
Semi-professional and public servant	86 (38.1)
Professional and officer	57 (25.2)
High-level professional and administrator	6 (2.7)
Household income^a^	
Low (<6,999 RMB per month)	58 (25.7)
Medium (≥7,000 and <19,999 RMB per month)	127 (56.2)
High (≥20,000 RMB per month)	41 (18.1)

a High, medium and low levels of household income were based on census data in the Fujian Statistics Yearbook (2022).

### 2.2. Procedure

A series of questionnaires were hand-delivered by the selected educators to the parents who agreed to participate. The questionnaires included the Demographics Questionnaire, the *Parenting Style and Dimension Questionnaire* (PSDQ), and the *Chinese Early Parental Involvement Scale* (CEPIS). Meanwhile, two trained graduate students who majored in early childhood education visited each kindergarten and assessed the participating children individually. The assessments were administered in a quiet room of the kindergarten they attended. Each child was tested with the *Picture Problem Solving Task* (PPST). The administration time lasted 10–15 min per child. After the test, the child was given a small gift and then sent back to his or her classroom.

### 2.3. Parent measures

#### 2.3.1. Parenting styles

The authoritative and authoritarian parenting styles were assessed using the 32-item *Parenting Style and Dimension Questionnaire* (PSDQ; Robinson et al., 2001). The PSDQ has been used worldwide (including parents of preschoolers in China) and linked to various child outcomes (e.g., Olivari et al., 2013; Xie and Li, 2019). In this study, Cronbach’s alphas for the authoritative parenting style scale (15 items) and authoritarian parenting style scale (12 items) were 0.90 and 0.87, respectively. Sample items in the authoritative parenting style scale include: “*Was responsive to child’s feelings or needs”* and “*Gave child reasons why rules should be obeyed.”* Sample items in the authoritarian parenting style scale include: *“Scolded and criticized child to make him/her improve”* and *“Punished child by taking privileges away from him/her with little if any explanations*.”

#### 2.3.2. Parental involvement

The *Chinese Early Parental Involvement Scale* (CEPIS; Lau et al., 2011) were employed to assess the extent to which parents were involved in six dimensions of informal home learning and formal kindergarten learning contexts: parent instruction (7 items), parent discussion (5 items), language and cognitive activities (5 items), homework (2 items), home-school conferencing (3 items), and school activities (4 items). Parents reported on their own involvement behaviors in the 26 items using a 5-point Likert scale. The CEPIS has shown good reliability and demonstrated as a significant predictor of Chinese preschoolers’ literacy and cognitive readiness (Lau et al., 2011). In this study, Cronbach’s alphas for dimensions of the CEPIS ranged from 0.77 (parent instruction) to 0.89 (language and cognitive activities). Sample items in the scale include: *“Play cognitively stimulating games together”* and *“Teach child to solve peer problems.”*

### 2.4. Child assessment

#### 2.4.1. Science skills

Science skills were assessed using the direct measure of children’s science-relevant problem-solving abilities using the *Picture Problem Solving Task* (PPST; Fusaro and Smith, 2018). In this task, seven pictures with real-world problematic scenarios were shown to an individual child one at a time. Sample items include: “*A strawberry is stuck frozen in an ice cube. What are ways to get the strawberry out?”* and “*Two bags are tied shut; What are ways to find out which one has pillows inside and which one has rocks inside, without opening them?.”* The assessor would describe the problem scenario briefly and ask the child to give solutions for solving the problem. The assessor would ask for additional solutions until the child had no more responses. Each child’s oral responses were immediately evaluated and scored based on the number of effective solutions. A good inter-rater reliability with the Cohen’s kappa of 0.81 was reached between two assessors for 25% of the child sample.

### 2.5. Data analyses

There were no missing responses in the dataset. IBM SPSS-25 was used to save and analyze all data. First of all, we conducted a Pearson’s correlation analysis among the variables before testing the hypotheses using IBM SPSS-25 program. Next, SPSS macro PROCESS 2.1 (Model 4) (Hayes, 2020) was conducted to test the mediating effect of parenting styles on children’s science problem-solving skills *via* parental involvement.

## 3. Results

### 3.1. Preliminary analyses

The results of Pearson’s correlation analysis (see Table 2) revealed that child gender was positively related to children’s science problem-solving skills. Results also revealed that authoritative parenting style was positively correlated with parental involvement, while authoritarian parenting style was negatively correlated with parental involvement. Parental involvement was positively correlated with children’s science problem-solving skills. Parenting styles were not correlated with children’s science problem-solving skills.

**Table 2:** Descriptive statistics and intercorrelations between variables.

	*M*	SD	1	2	3	4	5
1. Child gender	–	–	1				
2. Authoritative parenting	4.09	0.48	−0.09	1			
3. Authoritarian parenting	2.43	0.57	0.11	−0.35^***^	1		
4. Parental involvement	3.59	0.56	−0.08	0.49^***^	−0.14^*^	1	
5. Science problem-solving	6.76	2.65	0.19^**^	0.06	0.09	0.19^**^	1

*N* = 226. Person’s correlation was used. Child gender is virtual code, “0” = Female, “1” = Male. Theoretical ranges of the variables: Authoritative parenting = 0–5; Authoritarian parenting = 0–5; Parental involvement = 0–5; Science problem-solving = 0–21. ^*^p < 0.05; ^**^p < 0.01; ^***^p < 0.001.

### 3.2. Testing for the proposed model

**Figure 1:**
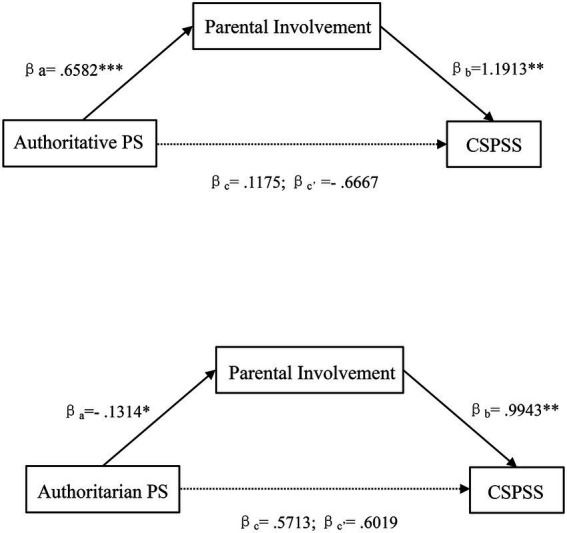
Mediation model of parenting styles on children’s science problem-solving skills *via* parental involvement. PS, parenting style; CSPSS, children’s science problem-solving skills. *p < 0.05; **p < 0.01; ***p < 0.001.

As shown in Figure 1 and Table 3, the authoritative parenting style was positively associated with parental involvement (*β* = 0.66, *p* < 0.001). A positive relationship between parental involvement and children’s science problem-solving skills was also found (*β* = 1.19, *p* < 0.01), after controlling for authoritative parenting. The results indicated parental involvement had a significant mediating effect in the relation between the authoritative parenting style and children’s science problem-solving skills, which supported H1a. Similarly, authoritarian parenting style had a significant negative association with parental involvement (*β* = −0.13, *p* < 0.05) and a significant positive relationship between parental involvement and children’s science problem-solving skills was also found (*β* = 0.99, *p* < 0.01), after controlling for authoritative parenting, thus supporting H1b.

**Table 3:** Mediation model of parenting styles on children’s science problem-solving skills *via* parental involvement.

	a	b	c’	c	ab	SE	LLCI ULCI
Authoritative PS → PI→ CSPSS	0.6582***	1.1913**	−0.6667	0.1175	0.7841*	0.3035	0.2296 1.4121
Authoritarian PS → PI→ CSPSS	−0.1314*	0.9943**	0.6019	0.5713	−0.1306*	0.0891	−0.3769

*N* = 226. The model was tested after controlling for child age, child gender, parent education and household income. PS, parenting styles; PI, parental involvement; CSPSS, children’s science problem-solving skills. *p < 0.05; **p < 0.01; ***p < 0.001.

**Figure 2:**
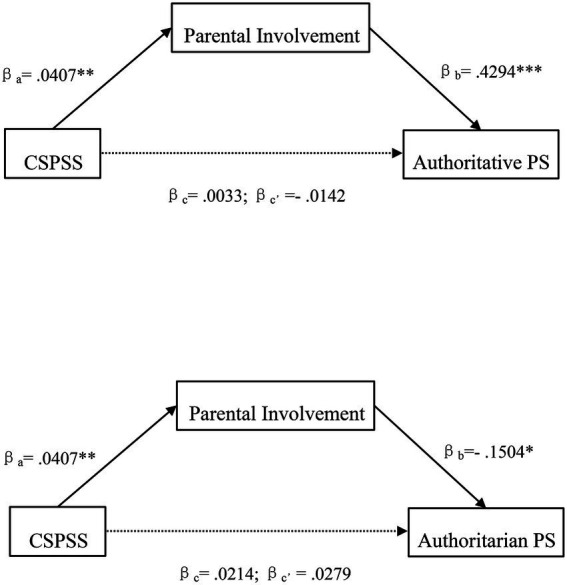
Mediation model of children’s science problem-solving skills on parenting styles *via* parental involvement. PS, parenting style; CSPSS, children’s science problem-solving skills. *p < 0.05; **p < 0.01; ***p < 0.001.

We also tested the mediating effect of children’s science problem-solving skills on parenting styles *via* parental involvement (See Figure 2). As shown in Table 4, a positive relationship between children’s science problem-solving skills and parental involvement was also found (*β* = 0.04, *p* < 0.01), after controlling for age, gender and family income. Also, there was a significant positive association between parental involvement and the authoritative parenting style (*β* = 0.43, *p* < 0.001). The results indicated parental involvement had a significant mediating effect in the relation between children’s science problem-solving skills and the authoritative parenting style, which supported H2a. Similarly, parental involvement had a significant negative association with the authoritarian parenting style (*β* = −0.15, *p* < 0.05), thus supporting H2b.

**Table 4:** Mediation model of children’s science problem-solving skills on parenting styles *via* parental involvement.

	a	b	c’	c	ab	*SE*	LLCI ULCI
CSPSS → PI→ Authoritative PS	0.0407**	0.4294***	−0.0142	0.0033	0.0175*	0.0067	0.0051 0.0315
CSPSS → PI→ Authoritarian PS	0.0407**	−0.1504*	0.0279	0.0214	−0.0065*	0.0041	−0.0169

*N* = 226. The model was tested after controlling for child age, child gender, parent education and household income. PS, parenting styles; PI, parental involvement; CSPSS, children’s science problem-solving skills. *p < 0.05; **p < 0.01; ***p < 0.001.

## 4. Discussion

Our evidence revealed that parental involvement has a significant mediating effect in the relation between both authoritative and authoritarian parenting styles and children’s science problem-solving skills. One key finding from our study was that the positive relationship between the authoritative parenting style and children’s science skills was mediated by the degree of parental involvement. This supplements Kashahu Xhelilaj et al.’s (2014) finding that authoritarian parents’ constant interference and demand accountability would annoy their children and create high levels of pressure on them, which distracts children from learning and ultimately results in reduced academic achievement. Our novel finding about the mediating role of parental involvement in linking parenting styles to children’s science skills contributes to the understanding of the processes through which parenting styles influence child development, which would continue to adolescence and even after adulthood.

Despite the theoretical argument that parenting styles influence child development by moderating the relationship between parenting practices and children’s developmental outcomes (Darling and Steinberg, 1993), this study provides evidence revealing that parental involvement, instead, would mediate the effects of parenting styles on young children’s science skills in China. This could be related to the particular cultural context in contemporary China, which shapes the interactions between parenting and early childhood learning and development (Lin et al., 2019). Chinese parents tend to promote children’s skills through their direct involvement (Lin et al., 2020b). Further cross-cultural research would benefit our understanding of the complex relationship between parenting styles and children’s science-relevant problem solving.

Another key finding of this study was that parental involvement also mediated the relation between children’s science problem-solving skills and parenting styles. This means that, in the families with a more responsive and demanding climate, the magnitude of parental involvement increased if the child had a higher level of science problem-solving skills, and vice versa. Unlike previous studies which investigated the links between parenting styles and parental involvement among adolescents’ families (e.g., Matejevic et al., 2014; Yang and Zhao, 2020), this study investigated Chinese young children’s parents and how the children’s science skills contributed to the relation between parenting styles and parental involvement. The bidirectional relationship between parenting styles, parental involvement and children’s science skills confirms that child participants may modify their parents’ expectations and actions (Kuczynski, 2003). It would be significant for future research to examine the links between the parenting context and other child outcomes using a bilateral conceptual framework for understanding dynamics in parent–child relations.

Overall, results of the present study corroborated and extended findings described in the Western research literature, with novel points revealed for understanding the processes of translating parenting styles to early childhood development, especially in the understudied domain – science skills development. The use of a bidirectional mediation model is a methodological contribution to understanding the mechanism underlying the relationship between parenting styles and child outcomes, although there are limitations, which are detailed below. Direct assessment of children’s science problem-solving skills was also an advantage of the present study due to its higher criterion validity and relevance to children’s authentic developmental status (Li et al., 2019).

## 5. Limitations and future directions

One of the limitations of this study is the reliance on self-report questionnaire data for examining parents’ parenting styles and parental involvement, which may increase the probability of inflated correlations (Bank et al., 1990). Future research could address such issues by using more direct approaches to evaluating parents’ parenting practices such as family practices rating scales and videotaped observations.

Also, the present study was conducted with a non-nationally representative sample from Fuzhou, China. In the future, it would be meaningful to replicate our research with a more representative sample in China or within cross-cultural contexts.

Last but not the least, this study relied upon a cross-sectional design in testing mediational processes, which fails to unveil the causal mediation effects. In addition, unmeasured confounding cannot be rejected. However, it provides the pilot data for testing the causal mediation effects using a longitudinal design. Future research can conduct mediation analyses with longitudinal data to explicitly test the temporal conditions.

## 6. Theoretical and practical implications

These findings have important implications for understanding the mechanism underlying the relation between parenting styles and children’s science skills. From a theoretical perspective, our findings support that parental involvement plays a mediating role in linking parenting styles and child outcomes, early science skills in particular. This adds to the integrative model of parenting style as context (Darling and Steinberg, 1993) by extending the moderating effect of parenting styles in understanding the parental influence. Moreover, our findings provide solid evidence regarding the positive effect of authoritative parenting style for creating a ‘positive emotional context’ (Berk, 2006, p. 567), which further promotes children’s early science problem solving and skills development.

From a practical perspective, these findings could support the design and implementation of family interventions for promoting children’s science reasoning, processing, and even integrated STEM skills. We come to know that authoritative parents may have positive impacts on children’s science learning in the early years, with their active involvement with children’s participation in early childhood education. In addition, parents adopting need supportive practices would strengthen the development of early child learning, which in turn enhances parents’ positive support and involvement (Moe et al., 2020). Therefore, practitioners should consider facilitating parents’ involvement and rational control in early learning and development through transforming their parenting styles to be more authoritative.

## 7. Conclusion

This study analyzed the mechanism underlying the relation between parenting styles and early science skills in Chinese young children. The results revealed that parental involvement was a significant mediator in linking parenting style to children’s science skills. The present study provides the first evidence to examine the role of parental involvement in linking parenting styles to children’s early science skills. It also contributes to the cultural diversity in researching the association between parenting context and child development by focusing on Chinese children and families.

## Data availability statement

The raw data supporting the conclusions of this article will be made available by the authors, without undue reservation.

## Ethics statement

The studies involving human participants were reviewed and approved by Research Ethics Committee of Education College, FNU. Written informed consent to participate in this study was provided by the participants’ legal guardian/next of kin.

## Author contributions

XL co-designed the research, collected the data, conducted the statistical analyses, and drafted the manuscript. WY determined the research questions and focus, co-designed the research, and drafted the manuscript. WX collected the data and edited the manuscript. HL provided important ideas and substantial feedback to the study and edited the manuscript. All authors contributed to the article and approved the submitted version.

## Funding

This project was supported by the National Education Sciences Planning Fund of China (Project No. BDA210076).

## Conflict of interest

The authors declare that the research was conducted in the absence of any commercial or financial relationships that could be construed as a potential conflict of interest.

## Publisher’s note

All claims expressed in this article are solely those of the authors and do not necessarily represent those of their affiliated organizations, or those of the publisher, the editors and the reviewers. Any product that may be evaluated in this article, or claim that may be made by its manufacturer, is not guaranteed or endorsed by the publisher.
